# A Comparison of Physical Activity Mobile Apps With and Without Existing Web-Based Social Networking Platforms: Systematic Review

**DOI:** 10.2196/12687

**Published:** 2019-08-16

**Authors:** Jasmine Maria Petersen, Ivanka Prichard, Eva Kemps

**Affiliations:** 1 College of Nursing and Health Sciences Flinders University Adelaide Australia; 2 College of Education, Psychology and Social Work Flinders University Adelaide Australia

**Keywords:** physical activity, mobile applications, social networking

## Abstract

**Background:**

Physical activity mobile apps present a unique medium to disseminate scalable interventions to increase levels of physical activity. However, the effectiveness of mobile apps has previously been limited by low levels of engagement. Existing Web-based social networking platforms (eg, Facebook and Twitter) afford high levels of popularity, reach, and sustain engagement and, thus, may present an innovative strategy to enhance the engagement, and ultimately the effectiveness of mobile apps.

**Objective:**

This study aimed to comparatively examine the effectiveness of, and engagement with, interventions that incorporate physical activity mobile apps in conjunction with and without existing Web-based social networking platforms (eg, Facebook and Twitter).

**Methods:**

A systematic review was conducted by following the Preferred Reporting Items for Systematic Reviews and Meta-Analysis Guidelines. A systematic search of the following databases was conducted: Medline, PsycINFO, Web of Science, Scopus, CINAHL, ProQuest, SPORTDiscus, EMBASE, and Cochrane. According to the comparative objective of this review, 2 independent literature searches were conducted. The first incorporated terms related to apps and physical activity; the second also incorporated terms related to Web-based social networking. The results of the two searches were synthesized and compared narratively.

**Results:**

A total of 15 studies were identified, 10 incorporated a physical activity app alone and 5 incorporated an app in conjunction with an existing Web-based social networking platform. Overall, 10 of the 15 interventions were effective in improving one or more physical activity behaviors. Specifically, improvements in physical activity behaviors were reported in 7 of the 10 interventions incorporating physical activity apps alone and in 3 of the 5 interventions incorporating physical activity apps in conjunction with existing Web-based social networking platforms. Interventions incorporating physical activity apps alone demonstrated a decline in app engagement. In contrast, the physical activity apps in conjunction with existing Web-based social networking platforms showed increased and sustained intervention engagement.

**Conclusions:**

The interventions incorporating physical activity apps in conjunction with and without existing Web-based social networking platforms demonstrated effectiveness in improving physical activity behaviors. Notably, however, the interventions that incorporated existing Web-based social networking platforms achieved higher levels of engagement than those that did not. This review provides preliminary evidence that existing Web-based social networking platforms may be fundamental to increase engagement with physical activity interventions.

## Introduction

Physical inactivity is a global pandemic. Globally, 1.4 billion adults (28%) are not meeting the physical activity guidelines (150 min of physical activity per week), a figure that is steadily increasing [1]. This is of public health concern given the consistently documented benefits of physical activity, including a reduced risk of cardiovascular disease, hypertension, osteoporosis, diabetes mellitus, obesity, mental illness, and premature mortality [2-4]. Thus, it is important to develop innovative, scalable interventions to increase levels of physical activity.

Advancements in mobile technology, specifically the development of mobile apps, present a unique medium to deliver interventions targeted at improving health behaviors. Mobile apps are software programs developed for mobile phones and tablets that hold potential to influence health behaviors owing to their widespread reach, accessibility, and convenience [5]. Recently, there has been a proliferation of mobile health apps, with estimates of over 318,000 available for download, double the number available 2 years ago [6]. Among mobile health apps, physical activity apps account for the largest proportion (30%) and are expected to increase 87% faster than any other category of health app [7]. Despite the ever-increasing ubiquity of physical activity mobile apps, previous reviews have only demonstrated modest evidence from such apps in terms of the magnitude of their effectiveness to positively influence physical activity behavior [8-11]. This indicates that there is potential to improve the effectiveness of physical activity mobile apps.

The effectiveness of mobile apps is influenced by levels of engagement with the app [8]. Specifically, a dose-response has been identified, such that increasing levels of engagement, and thus greater exposure to intervention content, is associated with improved behavioral outcomes [12]. Unfortunately, commercial research has identified a lack of commitment to sustained engagement with health and physical activity apps, reporting that few individuals (10%) engage with downloaded apps for more than 7 days [13,14]. An initial review of interventions incorporating physical activity apps also revealed rapid declines in app engagement over intervention periods of 3 and 9 months [15]. A more recent review further documented that interventions incorporating apps were effective only in the short term (<3 months), and this was purportedly linked to declining levels of engagement over time [11]. This is concerning given that long-term engagement in physical activity behaviors is important to attain any associated health benefits [16]. It is clear that strategies are needed to enhance engagement with mobile apps targeted at increasing physical activity. This, however, requires a greater understanding of the specific features of mobile apps that may augment engagement, and ultimately enhance their effectiveness.

An important consideration in the endeavor to improve the effectiveness of physical activity mobile apps is the appropriate utilization of behavior change theory. This is fundamental as the existing empirical literature has consistently identified that effective physical activity interventions are informed by theory [17,18]. However, previous research within the realm of physical activity interventions incorporating mobile apps has documented that the utilization of behavior change theory is largely lacking [19-22]. In addition, among the physical activity apps that are informed by theory, a diverse range of theories have been utilized including the Health Belief Model; Transtheoretical Model; Self-determination Theory; and Social Cognitive Theory [19-22]. This has limited the formation of conclusions regarding the most appropriate theoretical foundation(s) to inform the development of apps [23].

Behavior change theories are important in isolating specific features to incorporate into an intervention that will effectively facilitate behavior change. Given this, it is not surprising that an emerging body of research examining the content of physical activity mobile apps has identified that apps are lacking in the inclusion of features underpinned by behavior change theory [19-22]. Nevertheless, the limited theory-driven research to date has identified one particular feature, namely social support, that has been consistently incorporated into physical activity mobile apps and is underpinned by a myriad of behavior change theories [19-22]. Social support is commonly integrated into apps via Web-based social networking, which allows individuals to construct a personal profile and connect with other users [21]. Web-based social networks incorporated into physical activity mobile apps have a range of functionalities, including features that allow users to share physical activity data, receive *likes* and comments on their behavior (facilitating social interactions), and thus foster the provision of social support [21].

Typically, social support has been documented as a fundamental component of health interventions delivered face to face and has been associated with increased intervention engagement [12,24] and sustained behavior change [25]. Although face-to-face interventions may effectively facilitate high levels of support through interpersonal interactions, several limitations including time, cost, and resource intensiveness may hinder the viability of such interventions. Web-based social networks overcome many of the barriers of face-to-face interventions and afford several advantages including greater accessibility of immediate and continuous support, anonymity, and wide reach. Additionally, Web-based social networks incorporated into Web-based interventions targeting weight-related outcomes (eg, body weight and body mass index [BMI]) have demonstrated that the support provided is comparable with that attained in face-to-face interventions [26]. Thus, it has been suggested that the support provided by Web-based social networks may emulate the interpersonal support achieved through face-to-face interventions [27]. Evidently, Web-based social networking may be valuable in facilitating the provision of social support and fundamental in enhancing intervention engagement and thus effectiveness.

Previous research has ascertained 2 types of Web-based social networks incorporated into health interventions: (1) health-focused social networks (ie, networks developed by a researcher or integrated into health apps allowing users to connect with other users), and (2) existing social networking platforms (eg, Facebook and Twitter) [28,29]. In total, 2 systematic reviews have examined interventions (predominately Web-based) targeting health behaviors, including obesity, physical activity, sexual health, and smoking cessation, that either incorporated or were exclusively delivered via Web-based social networks (health-focused and existing) [28,29]. These reviews demonstrated positive effects of Web-based social networking in modifying health behaviors [28,29]. However, neither review [28,29] was able to identify the differing effectiveness of health-focused and existing Web-based social networks on influencing health outcomes and levels of engagement, as the 2 types of social networks were not evaluated independently. Notably, in both reviews, it was proposed that the inherent nature of existing Web-based social networking platforms may be harnessed to address issues of engagement and reach, ultimately enhancing the effectiveness of health interventions [28,29].

A recent meta-analysis [30] of interventions (eg, Web-based, face-to-face, and text messaging) targeting weight-related behaviors (eg, physical activity) and body weight status (eg, BMI) that either incorporated or were exclusively delivered via existing Web-based social networking platforms reported that these interventions produced significant reductions in body weight, BMI, and waist circumference, and significantly increased the average number of daily steps. This demonstrates that interventions incorporating, or exclusively delivered via existing Web-based social networking platforms, have the capacity to effectively modify a range of health-related outcomes. This may be attributed to the unique nature of existing Web-based social networking platforms, including their enormous popularity and widespread reach, with over 2.46 billion users worldwide, a figure that is continuing to rise [31]. Additionally, existing Web-based social networking platforms achieve high levels of sustained engagement, with estimates that 76% of Facebook users log in daily, 51% engage multiple times per day, and 70% continue to use the platform after 24 months [31]. Therefore, interventions that incorporate existing Web-based social networking platforms may achieve heightened effectiveness in their capacity to reach large audiences and sustain high levels of engagement.

Previously, no review has exclusively examined the effectiveness of interventions that incorporate physical activity mobile apps in conjunction with existing Web-based social networking platforms. The high prevalence of physical activity mobile apps, coupled with the promising capabilities of existing Web-based social networking platforms to augment app effectiveness, highlights an important avenue that warrants examination. Thus, this review examined the influence of existing Web-based social networking platforms on the effectiveness of, and engagement with, mobile apps that target physical activity. To isolate the influence of existing Web-based social networking platforms, this review provides a comparison between interventions that incorporate physical activity mobile apps in conjunction with and without existing Web-based social networking platforms.

## Methods

### Overview

**Figure 1:**
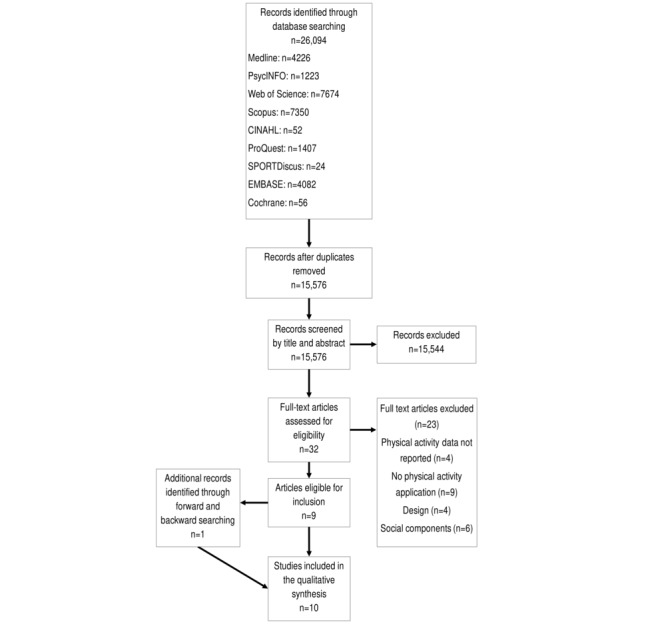
Preferred Reporting Items for Systematic Reviews and Meta-Analyses (PRISMA) flowchart: App-alone search.

**Figure 2:**
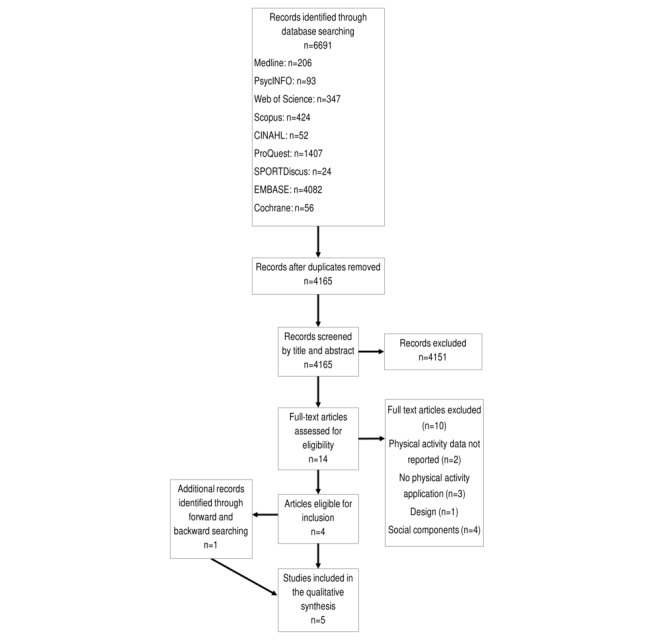
Preferred Reporting Items for Systematic Reviews and Meta-Analyses (PRISMA) flowchart: App Web-based Social Networking Search.

The systematic review was conducted according to the Preferred Reporting Items for Systematic Reviews and Meta-Analyses (PRISMA) Guidelines [32] (see Figures 1 and 2) and was registered with the International Prospective Register of Systematic Review (registration number CRD42018106456). An academic health librarian assisted with the development of the search strategy. The search strategy incorporated key terms and thesaurus terms related to mobile apps (eg, application, app, mobile phone, and iPhone), physical activity (eg, exercise, fitness, sports, inactive, and sedentary behavior) and Web-based social networks (eg, social network, social medium, Facebook, Twitter, and Instagram; see Multimedia Appendices 1 and 2 for complete search strategy). However, according to the comparative aims of this review, 2 independent searches were conducted, which differed such that one incorporated the terms related to apps and physical activity (app-alone search) and the other also incorporated the terms related to Web-based social networking (app Web-based social networking search). Both searches were conducted on the July 3, 2018, using the following 9 databases: Medline, PsycINFO, Web of Science, Scopus, CINAHL, ProQuest, SPORTDiscus, EMBASE, and Cochrane. The search results were limited to the English language, peer-reviewed, and year of publication between 2007 (the year smartphones were introduced) and the July 3, 2018.

### Inclusion Criteria and Study Selection

Studies from the 2 independent searches were selected if (1) a mobile app was incorporated as the main component of the intervention; (2) the primary or secondary outcome was to promote physical activity; (3) physical activity outcomes were reported; and (4) baseline and postintervention assessments of physical activity outcomes were included. The inclusion criteria differed slightly between the 2 searches to fulfill the comparative aims of the review. Specifically, the first search, termed app-alone, attempted to exclusively isolate the effect of physical activity apps, such that studies were deemed relevant if they did not incorporate any type of Web-based social network (health-focused or existing) or social component. Conversely, to ascertain the additive effects of an existing Web-based social network over and above that of an app, the second search, termed app Web-based social networking, required studies to specifically incorporate an existing Web-based social networking platform (eg, Facebook and Twitter) into their design. Included studies utilized an experimental or within-subjects pre-post design to determine the effectiveness of the intervention. Studies incorporating populations capable of engaging in physical activity were eligible for inclusion. In total, 2 reviewers independently screened the titles, abstracts, and full-text papers for eligibility and any disagreements were resolved by discussion. Forward (screening the citations of included studies) and backward (screening the reference lists of included studies) searching was conducted to ensure all relevant publications were identified.

### Data Extraction

Data extraction was conducted by the first author using a standardized form developed for this review. Extracted information included sample characteristics, study design, features of the mobile app, details of the Web-based social network, physical activity outcomes (time points reported), any additional outcomes reported (eg, engagement and psychosocial outcomes), and behavior change theories reported.

### Reporting of Methodological Characteristics

A 25-item tool devised by Maher et al [28] based on the Consolidated Standards of Reporting Trials (CONSORT) checklist [33] that examines reported methodological characteristics was used to assess methodological risk of bias. The tool was deemed to be relevant for this study as most of the items (20 out of 25) were applicable to both pre-post designs and randomized controlled trials [11,28]. The checklist was scored according to the extent to which each item was (1) fulfilled; (0.5) partially fulfilled; and (0) not fulfilled. A higher score is indicative of a lower risk of bias. In total, 2 independent reviewers assessed all included studies, and any disagreements were discussed and resolved.

### Data Synthesis

The primary outcome was physical activity behavior. The secondary outcomes included engagement with the intervention and psychosocial outcomes related to physical activity. In line with the comparative aims of the review, the app-alone and app Web-based social networking studies were compared in relation to both the primary and secondary outcomes. To determine whether the interventions effectively improved physical activity behavior, *P* values were evaluated. Specifically, interventions that were randomized controlled trials were identified to be effective if significant differences between groups across time were reported. Interventions of a pre-post study design were identified to be effective if significant changes across time were reported. Effect sizes were also examined and taken into account when evaluating the effectiveness of the interventions. The benchmark criteria for effect sizes are 0.20 for a small effect, 0.50 for a medium effect, and 0.80 for a large effect [34].

## Results

### Study Selection

The first database search (app-alone) identified 15,576 studies, following the removal of duplicates. Title and abstract screening deemed 15,544 studies ineligible for inclusion. In total, 32 full-text articles were screened for inclusion, with 23 studies excluded at this point (see Figure 1 for reasons). Forward and backward searching identified 1 additional study that was eligible for inclusion. A total of 10 app-alone studies were deemed relevant according to the predefined criteria and thus were included in this review (Figure 1).

The second database search (app Web-based social networking) identified 4165 studies, after removing duplicates. Title and abstract screening identified 4151 ineligible studies. In total, 14 full-text articles were screened for inclusion, resulting in 10 studies being excluded (see Figure 2 for reasons). Screening of reference lists and forward searching identified 1 additional study that was eligible for inclusion. A total of 5 studies were deemed suitable to be included in this review (Figure 2).

Thus, the following review included a total of 15 studies. Of these, 10 studies used an app alone, and 5 studies incorporated an app in conjunction with an existing Web-based social networking platform. These numbers of studies are similar to those of a recent comparative review [35].

### Characteristics of Included Studies

The characteristics of the app-alone studies are tabulated in Multimedia Appendix 3 and those of the app Web-based social networking studies are tabulated in Multimedia Appendix 4. The app-alone and app Web-based social networking studies were comparable in years of publication and the countries where the studies were conducted. However, the study designs differed such that the app-alone studies predominately utilized an experimental design (*n*=7) [36-42], whereas the app Web-based social networking studies predominantly utilized within-subjects pre-post designs (*n*=4) [43-46]. Across the 7 app-alone studies that utilized an experimental design, the control groups received either a no intervention control (*n*=1) [40]; minimal intervention (eg, accelerometer or print materials; *n*=5) [36-39,42]; or an app that differed slightly (fewer features; *n*=1) [41]. In contrast, the 1 app Web-based social networking study that included a control utilized a waitlisted control condition [47]. Among all included studies, 2 app Web-based social networking studies [45,47] aimed to modify physical activity in conjunction with dietary quality. Across the app-alone and app Web-based social networking studies, a greater number of interventions utilized newly designed apps (*n*=10) [40-45,47-50] than commercially available apps (*n*=5) [36-39,46]. The app-alone and app Web-based social networking studies incorporated samples that were similar in size, age, and the predominance of female participants. The samples that were composed of women, were women who were healthy [37,39,43], overweight and obese [47,48,50], insufficiently active [49,50], or nurses [44,45]. Although both the app-alone and app Web-based social networking studies largely recruited from a specific population (*n*=11) [38,40-42,44-50], disparities were noted among the app-alone and app Web-based social networking studies in relation to the populations recruited. Specifically, the app-alone interventions recruited samples that were sedentary (*n*=3) [38,40,49], low active (*n*=3) [41,42,50], obese or overweight (*n*=2) [48,50], in primary care (*n*=1) [36], pregnant (*n*=1) [38], or diagnosed with type 2 diabetes (*n*=1) [49]. Contrastingly, the app Web-based social networking interventions targeted samples that were nurses (*n*=2) [44,45], breast cancer survivors (*n*=1) [46], and obese or overweight (*n*=1) [47]. The average intervention duration for app-alone studies ranged from 1 week [40] to 14 weeks [50], comparable with the intervention durations of the app Web-based social networking studies that ranged from 3 weeks [44] to 3 months [45]. One app-alone study incorporated a 3-month follow-up assessment [42], whereas 2 app Web-based social networking studies incorporated follow-up assessments at 1 week postintervention [46] and 6 months postintervention [45].

Among the app-alone and app Web-based social networking studies, all apps targeted aerobic physical activity including light physical activity (*n*=6*)* [39,42,45,46,48,49], moderate physical activity (*n*=2) [39,42], moderate-to-vigorous physical activity (MVPA; *n*=6) [41,45-49], vigorous physical activity (*n*=2) [39,42], and daily steps (*n*=9) [36-38,42-46,50]. The apps incorporated a diverse range of features targeted at encouraging physical activity, including monitoring or tracking of behavior (*n*=9) [36,37,41,43,44,46-48,50], feedback (*n*=7) [36-38,40-43], information or education relating to physical activity (*n*=4) [38,40,41,47], goal setting (*n*=5) [41-43,45,50], and reinforcements (*n*=4) [40,41,48,50]. Both the app-alone and app Web-based social networking studies were underpinned by a diverse range of behavior change theories, namely the Social Cognitive Theory [38,40,41,43,45,46,50], Self-determination Theory [39], Control Theory [40,45], Goal-Setting Theory [45], attitude-social influence self-efficacy model [42], the Behavior Change Wheel [37], and the Theory of Reasoned Action [43].

### Description of the Existing Web-Based Social Networks

Among the app Web-based social networking studies, all 5 incorporated Facebook as the existing Web-based social networking platform; however, this platform was differentially utilized. In total, 2 studies provided participants with a link to a private Facebook group [45,47]; and 1 study incorporated a public Facebook page that included educational tips related to physical activity and participants were encouraged to comment and generate posts [46]. Alternatively, in 2 studies, the app had the functionality to connect to Facebook, whereby participants could share their physical activity data and receive *likes* and comments [43,44]. The existing Web-based social networks most often utilized features that facilitated social interaction (sharing physical activity posts, liking or commenting on others posts, and communicating with others; *n*=5) [43-47], social comparison (viewing posts of others’ physical activity performance; *n*=3) [43,44,46], and competition (ranking table and group averages; *n*=2) [43,44].

### Measures of Physical Activity and Additional Outcomes

Both the app-alone and app Web-based social networking studies primarily measured physical activity objectively (*n*=14) [36-38,40-50], specifically by utilizing an accelerometer (*n*=8) [41-43,45-49], pedometer (*n*=*3*) [36,37,44], Fitbit (*n*=2) [38,50] or inclinometer (*n*=1) [40]. Among all included studies, 2 app-alone studies measured physical activity by self-report, specifically by using the International Physical Activity Questionnaire (IPAQ)-Long form [42] and IPAQ-Short form [39]. Physical activity outcomes predominantly targeted for modification included light physical activity (*n*=6*)* [39,42,45,46,48,49], moderate physical activity (*n*=2) [39,42], MVPA (*n*=6) [41,45-49], vigorous physical activity (*n*=2) [39,42], daily steps (*n*=9) [36-38,42-46,50], or sedentary behavior (*n*=5) [40,45,46,48,49]. Across all studies, the underlying psychosocial outcomes related to physical activity (ie, self-efficacy and exercise motivation) were assessed by 4 app-alone studies [38,39,41,42] and 2 app Web-based social networking studies [45,46].

### The Effectiveness of the Intervention

Table 1 provides a summary of the intervention effects on physical activity outcomes. Across all included studies, 10 of the 15 interventions effectively improved one or more physical activity behaviors [36,37,40,41,43,44,46,48-50], including 7 of the 10 app-alone interventions [36,37,40,41,48-50] and 3 of the 5 app Web-based social networking interventions [43,44,46]. Improvements were reported in either the intervention conditions relative to a control condition (*n*=3) [36,37,40] or over time (*n*=7) [41,43,44,46,48-50] for one or more physical activity behaviors. Specifically, the physical outcomes reported were increases in daily steps (*n*=6) [36,37,43,44,46,50]; increases in light physical activity (*n*=2) [48,49]; increases in MVPA (*n*=3) [41,46,48]; and decreases in sedentary behavior (*n*=3) [40,46,48]. In total, 5 studies, 3 app-alone studies [38,39,42] and 2 app Web-based social networking studies [45,47], did not find an intervention effect across groups [38,39,42,47] or across time [45] in any of the physical activity behaviors measured. Effect sizes varied widely among both the app-alone and app Web-based social networking studies. Across the app-alone studies, effect sizes were small (*n*=2) [36,37], medium (*n*=2) [40,41], and large (*n*=1) [40]. Similarly, the distribution of effect sizes reported among the app Web-based social networking studies ranged from small (*n*=2) [45,46] to medium (*n*=2) [44,46] to large (*n*=1) [46].

Table 2 provides a summary of the intervention effects on psychosocial outcomes. The app-alone and app Web-based social networking studies overall reported mixed results in relation to psychosocial outcomes associated with physical activity. Specifically, 2 app-alone studies [39,42] and 1 app Web-based social networking study [45] revealed no significant intervention effects on any of the assessed psychosocial outcomes. In total, 2 app-alone studies reported significant decreases in perceptions of barriers to exercising in the intervention condition; however, not in the alternative outcomes assessed (eg, perceived social support and self-efficacy) [38,41]. Contrastingly, 1 app Web-based social networking study reported improvements over time in all psychosocial outcomes assessed (eg, social support, physical activity self-efficacy, and enjoyment) [46].

**Table 1:** Summary of intervention effects on physical activity outcomes.

Study		Physical activity (PA) outcomes			Engagement
		Daily steps	Light, moderate, moderate-to-vigorous physical activity (MVPA), and vigorous PA	Sedentary behavior	
**App-alone studies**					
	Arrogi et al, 2017 [40]	—^a^	—	[++]^b^	—
	Bond et al, 2014 [48]	—	[+]^c^	[+]	—
	Choi et al, 2016 [38]	[-]^d^	—	—	x^e^
	Cowdery et al, 2015 [39]	—	[-]	—	—
	Fanning et al, 2017 [41]	—	[+]	—	x
	Glynn et al, 2014 [36]	[++]	—	—	—
	Korinek et al, 2018 [50]	[+]	—	—	—
	Pellegrini et al, 2015 [49]	—	[+/−]^f^	[-]	✓^g^
	Simons et al, 2018 [42]	—	[-]	—	x
	Walsh et al, 2016 [37]	[++]	—	—	—
**App Web-based social networking studies**					
	Al Ayubi et al, 2014 [43]	[+]	—	—	✓
	Foster et al, 2010 [44]	[+]	—	—	✓
	Hurkmanns et al, 2018 [47]	—	[-]	—	—
	Pope et al, 2018 [46]	[+]	[+]	[+]	✓
	Torquati, Kolbe-Alexander et al, 2018 [45]	[-]	[-]	[-]	x

^a^Not applicable.

^b^Significant between-group improvement in outcome.

^c^Significant within-group improvement in outcome.

^d^No improvement in outcome.

^e^Unfavorable (low) engagement.

^f^Mixed results; engagement.

^g^Favorable (high) engagement.

**Table 2:** Summary of intervention effects on psychosocial outcomes.

Study		Psychosocial outcomes								Behavior change theories
		Social support	PA^a^ self-efficacy	PA motivation	Barriers to PA	PA enjoyment	Outcome expectations	Perceived benefits of PA	Perceived PA competency	
**App-alone studies**										
	Arrogi et al, 2017 [40]	—^b^	—	—	—	—	—	—	—	SCT^c^, CT^d^
	Bond et al, 2014 [48]	—	—	—	—	—	—	—	—	—
	Choi et al, 2016 [38]	[-]^e^	[-]	—	[++]^f^	—	—	—	—	SCT
	Cowdery et al, 2015 [39]	—	—	[-]	—	[-]	—	—	[-]	SDT^g^
	Fanning et al, 2017 [41]	—	[-]	—	[+]^h^	—	[-]	—	—	SCT
	Glynn et al, 2014 [36]	—	—	—	—	—	—	—	—	—
	Korinek et al, 2018 [50]	—	—	—	—	—	—	—	—	SCT
	Pellegrini et al, 2015 [49]	—	—	—	—	—	—	—	—	—
	Simons et al, 2018 [42]	[-]	[-]	—	[-]	—	—	[-]	—	ASE^i^
	Walsh et al, 2016 [37]	—	—	—	—	—	—	—	—	COM-B^j^
**App Web-based social networking studies**										
	Al Ayubi et al, 2014 [43]	—	—	—	—	—	—	—	—	SCT, TRA^k^
	Foster et al, 2010 [44]	—	—	—	—	—	—	—	—	—
	Hurkmanns et al, 2018 [47]	—	—	—	—	—	—	—	—	—
	Pope et al, 2018 [46]	[+]	[+]	—	—	[+]	—	—	—	SCT
	Torquati, Kolbe-Alexander et al, 2018 [45]	[-]	[-]	—	—	—	—	—	—	SCT, GST^l^, CT

^a^PA*:* physical activity.

^b^Not applicable.

^c^SCT: Social Cognitive Theory.

^d^CT: Control Theory.

^e^No improvement in outcome.

^f^Significant between-group improvement in outcome.

^g^SDT: Self-Determination Theory.

^h^Significant within-group improvement in outcome.

^i^ASE: Attitude-social Influence Self-efficacy Model.

^j^COM-B: The Capability, Opportunity, Motivation, Behavior framework.

^k^TRA: The Theory of Reasoned Action.

^l^GST: Goal setting Theory.

### Measures of Engagement

Notably, only 4 of the 10 app-alone studies (40%) reported on app usage [38,41,42,49], whereas 80% (*n*=4) of the app Web-based social networking studies assessed engagement with intervention materials (app and Web-based social network) [43-46]. Among the studies that assessed app engagement, objective measures were primarily utilized (*n*=6) [38,41-44,49]. This included the use of Google Analytics to monitor app logins and duration of use (*n*=2) [42,44], the functionality of the app to record logins (*n*=1) [41] or days and minutes of use (*n*=2) [43,49], or monitoring of engagement with app content (eg, reading or responding to automated messages and logging in activity diary; *n*=2) [38,42]. Self-report measures of app engagement were also utilized in 2 app Web-based social networking studies [45,46]. This included questionnaires whereby participants were asked to report frequency and duration of app use (*n*=1) [46] or engagement with app content (eg, willingness to use app and follow instructions; *n*=1) [45]. All studies that measured app engagement objectively (*n*=6) [38,41-44,49] monitored app usage over the duration of the intervention period. Conversely, among the 2 studies that utilized self-report measures, the questionnaires were completed at 2 time points: at mid and postintervention [46] and at postintervention and 6-month follow-up [45]. Among the app Web-based social networking studies, 2 reported engagement with the existing Web-based social network, such that the number of Facebook posts generated and posts viewed was monitored [45,46].

### Engagement With the Intervention

Among the 4 app-alone studies that assessed engagement with the app, 1 reported that, on average, the app was used on 21 days for a cumulative total of 7.6 hours, over a 1-month intervention period [49]. The other 3 studies reported a notable decline in app engagement [38,41,42]. Specifically, decreases were reported in the frequency and duration of app usage [41,42] and engagement with app content (logging physical activity and reading or responding to messages) [38,42] over 9-week [42] and 12-week intervention periods [38,41]. Among the app Web-based social networking studies, a single study reported limited engagement with the intervention materials over a 3-month intervention period, reporting that 68.4% of participants used the app less than once a month or never and 47.5% of participants engaged with the Facebook page on only one occasion per week [45]. Conversely, 2 reported increases in minutes of app usage following the provision of access to the existing Web-based social network [43,44], and 1 reported sustained engagement with intervention materials (app and Facebook page; *n*=1) [46].

### Comparison of Effective and Ineffective Interventions

As can be seen in Table 1, across all included studies, 7 of the 10 app-alone interventions (70%) [36,37,40,41,48-50] and 3 of the 5 app Web-based social networking interventions (60%) [43,44,46] were effective in improving one or more physical activity behaviors, as identified by *P* values and/or effect sizes. Among the effective interventions, the intervention durations were relatively short, ranging from 1 week [40] to 14 weeks [50]. In comparison, the ineffective interventions typically incorporated longer intervention durations, ranging from 9 weeks [42] to 3 months [45]. Notably, 6 of the 10 (60%) effective interventions recruited low-active (*n*=2) [41,50] or sedentary participants (*n*=2) [40,49], or documented that participants engaged in low levels of baseline physical activity (*n*=2) [37,48]. By contrast, only 2 of the 5 (40%) ineffective interventions recruited low-active (*n*=1) [42] or sedentary participants (*n*=1) [38]. The effective interventions all exclusively targeted physical activity behaviors. The 2 app Web-based social networking interventions that were not effective [45,47] both targeted the modification of physical activity in conjunction with diet quality. Across all included studies, objective measures of physical activity were predominately utilized (*n*=14) [36-38,40-50], and the type of objective measure used (eg, accelerometer) was comparable among the effective and ineffective interventions. However, 2 of the 5 ineffective interventions utilized self-report measures to assess the physical activity behaviors [39,42]. Both the effective (*n*=6) [37,40,41,43,46,50] and ineffective (*n*=4) [38,39,42,45] interventions were largely underpinned by behavior change theories. Among the 10 effective studies, 7 (70%) used newly designed apps [40,41,43,44,48-50] and 3 (30%) used commercially available apps [36,37,46]. Among the 5 ineffective studies, 3 (60%) used newly designed apps [42,45,47] and 2 (40%) used a commercially designed app [38,39].

In total, 2 of the effective interventions [41,46] assessed psychosocial outcomes, and mixed findings were reported. Specifically, the app-alone study that incorporated a newly designed app reported no changes in physical activity self-efficacy or physical activity outcome expectancies but identified a decrease in perceptions of barriers to exercising [41]. In contrast, the app Web-based social networking study that incorporated a commercially available app reported increases in physical activity self-efficacy, physical activity enjoyment, and social support [46]. In total, 4 of the ineffective studies assessed psychosocial outcomes [38,39,42,45], and although 1 study identified a decrease in the lack of energy as a barrier to exercising [38], no changes were reported in any of the alternative outcomes assessed, including social support [38,42,45], physical activity self-efficacy [38,42,45], physical activity enjoyment [39], physical activity motivation [39], perceived competency for exercising regularly [39], and perceived benefits to exercising [42].

Among the effective studies, 1 app-alone study [41] and all app Web-based social networking studies (*n*=3) [43,44,46] reported on app engagement. The app-alone study reported a decline in app usage over the 12-week intervention period [41]. In contrast, in the app Web-based social networking studies, higher app usage following the provision of access to the Web-based social networking functionalities [43,44] and sustained engagement with intervention materials (app and Facebook page) were reported [46]. Among the ineffective studies, 3 of the 5 studies reported on intervention engagement [38,42,45]. Of these studies, all reported unfavorable intervention engagement, specifically declines in app engagement during a 9-week [42] and 12-week intervention period [38], and low engagement with intervention materials (app and Facebook group) [45]. Additionally, among the effective app Web-based social networking interventions, the existing social networks utilized were a public Facebook page (*n*=1) [46] or a physical activity app that incorporated functionalities to connect with Facebook (*n*=2) [43,44]. Among the 2 ineffective app Web-based social networking interventions, both incorporated a private Facebook group as the existing Web-based social network [45,47].

### Reporting of Methodological Characteristics

The reported methodological characteristics were examined to generate a methodological risk of bias score. Scores ranged from 9.5 (out of 20) to 20.5 (out of 25) in the app-alone studies (Multimedia Appendix 5) and from 8.5 (out of 20) to 18 (out of 25) in the app Web-based social networking studies (Multimedia Appendix 6). The app-alone and app Web-based social networking studies all fulfilled the checklist criteria for scientific background and a detailed description of the intervention. Among the randomized controlled trials (*n*=8), few adequately reported on the allocation concealment mechanisms (*n*=3) [36,41,42] or blinding (*n*=3) [36,41,47]; however, most did report on randomization procedures (*n*=7) [36-39,41,42,47]. Notably, both the app-alone and app Web-based social networking studies rarely fulfilled the criterion detailing how the sample size was calculated (*n*=8) [36,37,39,41-43,47,48] or appropriately reported on the study outcomes (effect sizes; *n*=7) [36-38,40,41,44,48].

## Discussion

### Principal Findings

This review examined the influence of existing Web-based social networking platforms on the engagement with, and effectiveness of, mobile apps that target physical activity. Specifically, to isolate the influence of existing Web-based social networking platforms, the review provided a comparison between interventions that incorporated physical activity apps in conjunction with and without existing Web-based social networking platforms.

The review identified that physical activity mobile apps show promise in their capacity to improve physical activity behaviors. Of the included studies, 10 of the 15 interventions effectively improved one or more physical activity behaviors [36,37,40,41,43,44,46,48-50]. Specifically, 7 of the 10 app-alone studies [36,37,40,41,48-50] and 3 of the 5 app Web-based social networking studies [43,44,46] reported improvements. At a surface level, these findings indicate that the app Web-based social networking interventions may be no more effective than the app-alone interventions. However, this may be attributed to methodological disparities between the app-alone and app Web-based social networking interventions rather than the presence of Web-based social networking *per se*. Specifically, heterogeneity in the recruited samples may have influenced physical activity outcomes and thus must be considered in the formation of accurate conclusions regarding intervention effectiveness. This is highlighted in the comparison of 2 app-alone [48,50] and an app Web-based social networking intervention [47] that all targeted the modification of physical activity in overweight or obese individuals. The 2 app-alone interventions [48,50] both improved physical activity levels, whereas the app Web-based social networking study did not [47]. However, both app-alone studies [48,50] reported low baseline levels of physical activity, which may have influenced intervention outcomes. Furthermore, the differences in the samples recruited may also be responsible for overall differences in intervention effectiveness between the app-alone and app Web-based social networking studies. Specifically, 80% (*n*=8) of the app-alone interventions recruited low-active (*n*=3) [41,42,50] or sedentary participants (*n*=3) [38,40,49] or reported that participants engaged in low levels of physical activity at baseline (*n*=2) [37,48]. Of these interventions, 75% (*n*=6) [37,40,41,48-50] reported improvements in physical activity behaviors. This is consistent with previous literature documenting that physical activity interventions demonstrate greater effectiveness among low-active individuals, as there is a larger potential for improvement in behavior [51]. In contrast, none of the app Web-based social networking interventions incorporated recruitment criteria regarding sedentary or physical activity behaviors or reported low baseline levels of physical activity. Thus, the disparity among the samples may have influenced intervention outcomes, limiting the formation of appropriate conclusions regarding the influence of existing Web-based social networking platforms on intervention effectiveness. Future research is needed to evaluate the effectiveness of apps in conjunction with Web-based social networks in low-active or sedentary populations.

The comparability of intervention engagement between the app-alone and the app Web-based social networking interventions is also somewhat limited by the lack of reporting on engagement in the app-alone studies. This is consistent with existing reviews that have documented a lack of assessment of engagement in interventions targeting health behaviors [8]. This presents a shortcoming of research to date, such that the previously limited assessment of engagement has hindered the identification of intervention components that may be associated with engagement. This review identified clear differences in the levels of engagement reported among the app-alone and app Web-based social networking studies. The app-alone studies that reported on patterns of engagement identified declines in app engagement over 9-week [42] and 12-week intervention periods [38,41]. Of these studies, 1 reported improvement in physical activity behaviors [41], whereas the other 2 did not [38,42]. Across the app Web-based social networking studies, 1 study reported low engagement with intervention materials (app and Facebook group), and notably no improvement in physical activity outcomes [45]. In contrast, all other app Web-based social networking studies reported increases in engagement following the provision of access to the existing Web-based social networking platform [43,44] and sustained engagement with intervention materials (app and Facebook page) [46]. Among these studies, all reported improvements in physical activity behaviors [43,44,46], in line with previous evidence linking engagement with intervention effectiveness [8,12]. Thus, the app-alone studies demonstrated the typically observed decline in app engagement [38,41,42], whereas the app Web-based social networking studies showed increased and sustained intervention engagement [43,44,46]. This review provides preliminary evidence that existing Web-based social networks may be an important component in increasing engagement with physical activity interventions.

The existing Web-based social networking platform incorporated into all the app Web-based social networking interventions was Facebook, including either a public Facebook page [46], a private Facebook group [45,47], or a physical activity app that had the functionality to connect to Facebook [43,44]. The existing Web-based social networks utilized a diverse range of features that primarily facilitated social interaction, social comparison, and competition. However, the heterogeneity in the features utilized, and the predominance of studies that incorporated several different features, limited the capacity to ascertain the association between specific features of Web-based social networking and app engagement. Interestingly, the findings indicated that the differential use of the Facebook platform may have influenced intervention effectiveness. The interventions incorporating a private Facebook group did not report improvements in physical activity behaviors [45,47]. Of these interventions, one study [45] reported on intervention engagement and psychosocial constructs, identifying low intervention engagement, and no changes in social support or self-efficacy. Contrastingly, the interventions that incorporated a Facebook page [46], or an app that connected with Facebook [43,44] showed improvements in physical activity behaviors and resulted in increased and sustained engagement. Additionally, increases were reported in social support, self-efficacy, and physical activity enjoyment in one of these studies [46]. Importantly, these are all psychosocial constructs associated with facilitating physical activity behaviors [25], intervention engagement [12,24], and sustained behavior change [25]. Notably, among the interventions that produced favorable outcomes [43,44,46], participants’ existing networks were leveraged via apps that connected with Facebook [43,44], or a Facebook page [46]. Contrastingly, the interventions that produced unfavorable outcomes [45,47] incorporated private Facebook groups that generated an artificial Web-based social network, such that participants were required to create connections with unknown others. This indicates that network dynamics may be an important underlying determinant of the influence of Web-based social networks on intervention outcomes.

### Implications for Future Research

This review suggests that the way in which Web-based social networking platforms are utilized must be considered in the development of interventions as it has important implications for intervention effectiveness. This highlights a gap in the literature, such that little guidance exists in relation to optimally harnessing Web-based social networking platforms in behavior change interventions. Future research must endeavor to identify specific features of Web-based social networking platforms that are associated with intervention engagement, to ascertain how best to incorporate Web-based social networking into health interventions. However, this will require a greater understanding of the mechanisms (eg, social support) underlying the influence of Web-based social networking on health behaviors, to elucidate how best to leverage specific features of Web-based social networking platforms in health interventions. In addition, Web-based social networking is evolving rapidly, and, thus, an understanding of the underlying mechanisms will be advantageous in identifying how to optimally leverage a diverse range of social networking platforms in future interventions.

The present review further ascertained disparities among the designs and quality of app-alone and app Web-based social networking studies. The app-alone interventions were predominately randomized controlled trials; by contrast, the app Web-based social networking studies were largely pre-post within-subjects designs. Thus, future research must endeavor to utilize study designs of a higher standard (ie, randomized controlled trials) to increase the quality of evidence pertaining to the effectiveness of interventions incorporating physical activity apps in conjunction with Web-based social networking. Furthermore, the app-alone and app Web-based social networking studies incorporated predominately short intervention durations, and across all studies in the review, only 3 included follow-up assessments, at 1-week postintervention [46], 3 months [42], and 6 months postintervention [45]. The dearth of evidence regarding the long-term efficacy of mobile apps is frequently documented as an important shortcoming. Evaluating the long-term effectiveness of mobile apps is imperative, as sustained engagement in physical activity behavior is required to attain the associated health benefits [16].

The review identified several features of the interventions that may be important in guiding the design of future interventions. Specifically, interventions that were effective targeted exclusively the modification of physical activity behaviors. This is consistent with previous research identifying that single behavior change interventions targeting physical activity are more effective than interventions that target multiple behaviors (eg, physical activity and dietary behavior) [52,53]. Although interventions that target multiple health behaviors simultaneously have the potential to maximize health benefits, evidence suggests that the modification of one behavior will enhance intervention outcomes [52,53]. Furthermore, the interventions that were effective incorporated objective measures of physical activity [36,37,40,41,43,44,46,48-50]. Interestingly, the 2 studies that incorporated a self-report measure of physical activity did not report an increase in physical activity over intervention periods of 9 [42] and 12 weeks [39]. It is possible that self-report measures as opposed to objective measures such as accelerometers afford lower sensitivity to detect changes in physical activity behaviors over short intervention periods [54]. Indeed, a previous review has demonstrated that 69% of studies that incorporated self-report measures, as opposed to 20% of studies that measured physical activity objectively, found no effect on physical activity [9]. In addition, in this review, comparatively, there was no difference in the effectiveness of interventions that used a newly designed app as opposed to a commercially available app. Despite this, the interventions largely utilized newly designed apps. This is problematic as commercially available apps are ubiquitous and highly accessible to the general public; however, evidence of their effectiveness is lacking [19,20,22]. Thus, future research should evaluate the effectiveness of commercially available physical activity mobile apps.

Overall, the mobile apps were effective in increasing physical activity in a diverse range of population samples, including inactive [41,50], sedentary [40,49], obese or overweight individuals [48,50], breast cancer survivors [46], and individuals diagnosed with type 2 diabetes [49]. However, all studies exclusively targeted adults, ranging from 20 [37] to 53 years [49]. Thus, future research must endeavor to evaluate the applicability of physical activity mobile apps in conjunction with existing Web-based social networks in alternative age groups, in particular among adolescents, a highly inactive population subgroup [55], and among the highest users of existing Web-based social networking platforms [56]. This will ensure that mobile apps are an appropriate medium to disseminate physical activity interventions that are scalable, owing to their applicability to the population broadly.

This review also has important implications for guiding the development of an appropriate theoretical foundation to inform future physical activity mobile apps. The included interventions incorporated mobile apps predominately underpinned by behavior change theory [37-43,45,46,50]. This suggests that there was no association between mobile app effectiveness and the utilization of any one particular theory. Additionally, across the included studies a diverse range of behavior change theories were utilized, limiting the formation of conclusions regarding the most effective theory to guide the development of physical activity mobile apps. This is consistent with previous research examining the content of physical activity mobile apps that has documented challenges ascertaining the theory or combination of theories associated with physical activity mobile app effectiveness [23].

The physical activity apps examined in this review incorporated a diverse range of features. The most common among these were monitoring or tracking of behavior, feedback, information or education related to physical activity, goal setting, and providing reinforcements (eg, points). Much of the previous research that has examined the content of physical activity apps has utilized a taxonomy developed by Abraham and Michie [57] that functions to isolate the presence of behavior change techniques common to many behavior change theories. This research has identified that feedback, self-monitoring, and goal setting are features frequently integrated into apps, in line with findings by this review [19,58,59]. Notably, Abraham and Michie [60] highlight that these features are also commonly associated with effectively modifying physical activity behavior. This may have underpinned the capacity of the majority of the apps in the current review to improve physical activity behavior. However, the specific number or combination of features that may have a greater influence on the effectiveness of physical activity apps is currently unknown, and thus requires future examination.

### Limitations

To our knowledge, this is the first review to isolate the influence of existing Web-based social networking platforms by providing a comparison between interventions that incorporate mobile physical activity apps in conjunction with and without existing Web-based social networking platforms. Despite the novel nature of this review, several limitations must be noted. First, to date, there are only a small number of studies that have incorporated physical activity mobile apps in conjunction with an existing Web-based social networking platform. Additionally, owing to the heterogeneity of the identified studies in relation to the target population, intervention, study design, and outcomes measured, the results could not be validly pooled, precluding the ability to conduct a meta-analysis, and, thus, form definitive conclusions regarding the influence of Web-based social networks. Second, all interventions incorporated apps that targeted aerobic activity, and, thus, the findings may not generalize to apps aimed at other types of physical activity such as strength training. Future research should endeavor to examine apps targeted at all forms of physical activity. Third, among the included studies the methodological risk of bias varied, with some studies receiving low scores, limiting the trust that may be placed in their findings. Finally, there is a possibility of publication bias as the search did not incorporate gray literature or non-English publications.

### Conclusions

In conclusion, the unprecedented growth in physical activity mobile apps presents an innovative medium to disseminate scalable interventions to increase levels of physical activity worldwide. However, previous literature has consistently documented that the effectiveness of mobile apps is limited by low levels of engagement. The popularity, reach, and engagement afforded by existing Web-based social networking platforms provides an unparalleled opportunity to serve as an adjunct to mobile apps to augment engagement, and ultimately effectiveness. Thus, this review aimed to provide insight into the influence of existing Web-based social networks by providing a comparison between interventions that incorporated mobile apps in conjunction with and without existing Web-based social networking platforms. Both the interventions incorporating physical activity apps in conjunction with and without existing Web-based social networking platforms demonstrated effectiveness in improving physical activity behaviors. Notably, however, interventions that incorporated existing Web-based social networking platforms achieved higher levels of engagement than those that did not. This provides preliminary evidence that existing Web-based social networking platforms may be fundamental in overcoming the previously documented low engagement associated with physical activity mobile apps. This is of particular importance as greater app engagement is associated with increased exposure to intervention content, and ultimately an enhanced capacity of the app to effectively improve physical activity behavior. Thus, existing Web-based social networks must be further evaluated by conducting rigorously designed randomized controlled trials. Importantly, future research must endeavor to provide a greater understanding of the mechanisms underlying the influence of Web-based social networking on physical activity behaviors, to ascertain how best to leverage specific features of Web-based social networking platforms. This review makes an important contribution to guiding future research, by providing an initial insight into mobile apps and existing Web-based social networking platforms, imperative to improving the development of interventions targeted at increasing physical activity levels.

## Appendix

Multimedia Appendix 1 App-alone search strategy.

Multimedia Appendix 2 App Web-based social networking search strategy.

Multimedia Appendix 3 Characteristics of app-alone studies.

Multimedia Appendix 4 Characteristics of app Web-based social networking studies.

Multimedia Appendix 5 Risk of bias based on Consolidated Standards of Reporting Trials Checklist; App-alone studies.

Multimedia Appendix 6 Risk of bias based on Consolidated Standards of Reporting Trials Checklist; App Web-based social networking studies.

## Abbreviations

BMI: Body Mass Index
CONSORT: Consolidated Standards of Reporting Trials
IPAQ: International Physical Activity Questionnaire
MVPA: moderate-to-vigorous physical activity
PRISMA: Preferred Reporting Items for Systematic Reviews and Meta-Analyses
