# Supplementary material for: A Comparison of Physical Activity Mobile Apps With and Without Existing Web-Based Social Networking Platforms: Systematic Review
Source: J Med Internet Res. 2019 Aug 16;21(8):e12687. doi: 10.2196/12687 (PMC6716337; doi:10.2196/12687)
Supplement: Multimedia Appendix 1 [file jmir_v21i8e12687_app1.pdf]

Physical activity component	1. Exp Exercise/<br><br>2. Exp Sports/<br><br>3. (exercis* or sport* or physical activit* or fitness or walk* or running or swim* or jogging or cycling or sedentar* or sedentary lifestyle or behav* change or inactiv* or gym* or basketball or baseball or hockey or racquet sports or soccer or volleyball or netball or football).ti,ab.<br><br>4. 1 or 2 or 3
Applications component<br><br><br><br><br><br><br><br><br><br>Limits	5. Mobile Applications/<br><br>6. Cell Phone/<br><br>7. Computers, Handheld/ or Smartphone/<br><br>8. (app or apps or mobile application* or iphone* or ipad* or smartphone* or cell phone* or cellphone* or android* or tablet* or handheld computer* or blackberry* or Itunes or mHealth or mobile health or mobile technolog* or mobile device* or electronic health or eHealth or digital intervention* or Sweatcoin or MyFitnessPal or Freeletics or Strava or Zombies, Run! or Nike + Run Club or Fitness Buddy or MayMyRun or RunKeeper or Runtastics). ti,ab.<br><br>9. 5 or 6 or 7 or 8<br><br>10. 4 and 9<br><br>English language<br>Peer-reviewed<br>Year of publication from 01/01/2007 to 03/07/2018.
