# Supplementary material for: A Comparison of Physical Activity Mobile Apps With and Without Existing Web-Based Social Networking Platforms: Systematic Review
Source: J Med Internet Res. 2019 Aug 16;21(8):e12687. doi: 10.2196/12687 (PMC6716337; doi:10.2196/12687)
Supplement: Multimedia Appendix 3 [file jmir_v21i8e12687_app3.pdf]

Reference	Country	Sample Characteristics	Study Design	Description of Intervention	Physical Activity Outcome Measure(s)	Other Measures	Key Findings	Behaviour Change Theory
Arrogi et al., 2017 [40]	United Kingdom	58 participants (51 analysed)<br><br>18-55 years<br><br>Mean age: 36.2 years (10.2)<br><br>41.10% females<br><br>Sedentary lifestyle	2-week, 2-group RCT<br><br>Pre-intervention assessments: 1-week	*Mobile application*<br>stApp (newly developed): Alarm alert after 25 and 30 minutes of prolonged sitting; tailored feedback based on sitting behaviour; information to reduce sitting behaviour; encouragement; motion sensor to detect sitting behaviour<br><br>*Intervention condition* (*n* = 28)<br>Received stApp; wore activPAL3 inclinometer<br><br>*Control condition* (*n* = 23)<br>Did not receive stApp; wore activPAL3 inclinometer	*Pre-intervention & 2 weeks*<br><br>activPAL3 inclinometer<br>Sitting behaviour		*Physical activity outcomes*<br><br>Significant group by time interaction for total sitting time on week days, $P = .03$, $d = 0.62$<br>Total sitting time significantly decreased in the intervention condition from baseline (M = 633.9, SD = 81.9) to post-intervention (M = 593.4, SD = 111.5) ($P = .002$, $d = 0.41$)<br>No significant difference in control condition from baseline (M = 658.4, SD = 74.4) to post-intervention (M = 658.1, SD = 66.0) ($P > .05$, $d = 0.00$)<br><br>Significant group by time interaction for prolonged sitting bouts on week days ($P < .001$, $d = 1.35$)<br>Significant decrease in prolonged sitting bouts on week days in the intervention condition from baseline (M = 6.2, SD = 1.6) to post-intervention (M = 3.4, SD = 2.0) ($P < .001$, $d = 1.5$)<br>No significant difference in prolonged sitting bouts on week days in control condition from baseline (M = 6.7, SD = 1.8) to	Social Cognitive Theory;<br>Control Theory

							post-intervention (M = 6.3, SD = 1.9 ($P > .05$, $d = 0.22$)	
Bond et al., 2014 [48]	USA	30 participants<br><br>21-70 years<br><br>Mean age: 47.5 years (13.5)<br><br>83.3% female<br><br>Overweight or obese (BMI = ≥ 25 kg/m^2)	28-days within-subject, pre-post design<br><br>Pre-intervention assessments: 1-week	*Mobile application*<br>B-MOBILE Application (newly developed):<br>Audible prompts and reminders based on break goal condition (see below); accelerometer to monitor sedentary behaviour; tracking of total sedentary and active minutes accumulated throughout the day; praise for compliance with physical activity break prompts<br><br>3 physical activity break goal conditions:<br>1. 3 minute break after 30 continuous minutes of sedentary behaviour<br>2. 6 minute break after 60 continuous minutes of sedentary behaviour<br>3. 12 minute break after 120 continuous minutes of sedentary behaviour<br><br>*Intervention*<br>10 minutes of in-person education at the start of the intervention on reducing sedentary behaviour; received B-MOBILE application; engagement in each of the 3 conditions for a 7-day period; wore SenseWear Mini Armband monitor	*Pre-intervention & during all 3 conditions*<br><br>SenseWear Mini Armband monitor Objective measure of sedentary behaviour, light and moderate-to-vigorous intensity physical activity (MVPA)		*Physical activity outcomes (M & SD not reported)*<br><br>Percentage of time spent in sedentary behaviour was significantly decreased in all 3 conditions relative to baseline ($P < .005$)<br><br>Percent time spent in light ($P < .05$) and MVPA ($P < .01$) significantly increased in all 3 conditions relative to baseline	Not Reported

Choi et al., 2016 [38]	USA	30 participants<br>18-40 years<br>Mean age 33.7 years (2.6)<br>100% female<br>Pregnant (10-40 weeks gestation) & sedentary lifestyle	12-week, 2-group RCT<br>Pre-intervention assessments: 1-2 weeks	*Mobile application*<br>Fitbit (commercially available):<br>Self-monitoring; displays steps, distance, flights of steps climbed and calories expended; automated daily messages to support physical activity; activity diary to report daily steps, type and duration of physical activity engaged in; feedback given on progress; tips for physical activity, healthy diet and weight management during pregnancy<br>*Intervention condition* (*n* = 15)<br>Received mobile application; initial in-person session: provision of information including; physical activity recommendations for pregnant women; goal setting; and safety instructions for increasing physical activity during pregnancy; wore a Fitbit Ultra<br>*Control condition (n* = 15*)*<br>Did not received mobile application; initial in-person session: recommendations for increasing physical activity during pregnancy and safety instructions for increasing physical activity during pregnancy; wore a Fitbit Ultra	*Pre-intervention & 12 weeks*<br>Fitbit Ultra<br>Weekly step counts	*Pre-intervention & 12 weeks*<br>Self-efficacy for Physical Activity<br>Social Support and Exercise Survey<br>App engagement<br>Responses to automated daily messages and logging of physical activity in application activity diary	*Physical activity outcomes*<br>No significant between-group change in mean daily steps, *P* = .23<br>*Psychosocial outcomes*<br>Significant between-group change in lack of energy as barrier to exercising, *P* = .02<br>Intervention condition; baseline (M = 5.13, SD = 2.56); post intervention (M = 3.62, SD = 2.90)<br>Control condition; baseline (M = 4.13, SD = 2.59); post-intervention (M = 4.80, SD = 2.08)<br>No significant between-group changes in self-efficacy (*P* = .58) or social support from family (*P* = .28) and friends (*P* = .64)<br>*Intervention engagement*<br>Decrease in responding to daily messages and logging of physical activity in activity diary (*P*-value not reported).<br>Insufficient data to calculate effect sizes	Social Cognitive Theory

Cowdery et al., 2015 [39]	USA	40 participants<br><br>18-69 years<br><br>Median age: 32 years<br><br>85% female	12-week, 2-group RCT<br><br>Pre-intervention assessments: Initial in-person session	*Mobile application*<br><br>Zombies, Run! (commercially available):<br>Immersive running game; audio adventure; player collects supplies and avoids being attacked by Zombies as they exercise<br><br>The Walk (commercially available):<br>Audio adventure game; episodes and challenges; tasked with a package that must be delivered in order to save the world<br><br>*Intervention condition* (*n* = 20)<br>Choice between one of the two applications; tracking app (MOVES); weekly motivational emails<br><br>*Control condition (n* = 20*)*<br>Did not receive a commercially available app*;* tracking app (MOVES)	*Pre-intervention & 12 weeks*<br><br>International Physical Activity Questionnaire (IPAQ)-Short Form<br>Weekly minutes of light, moderate and vigorous physical activity	*Pre-intervention & 12 weeks*<br><br>Physical Activity Enjoyment Scale<br><br>Treatment Self-Regulation Questionnaire for exercise;<br>Autonomous motivation, controlled motivation and amotivation<br><br>Perceived Competence for Exercising Regularly Scale	*Physical activity outcomes*<br><br>No significant group by time interaction for light (*P* = .32) moderate (*P* = .57) or vigorous physical activity (*P* = .87)<br><br>*Psychosocial outcomes*<br><br>No significant group by time interaction for physical activity enjoyment (*P* = .66) autonomous motivation (*P* = .92), controlled motivation (*P* = .69), amotivation (*P* = .16) or perceived competence (*P* = .06)<br><br>Insufficient data to calculate effect sizes	Self-determination Theory
Fanning et al., 2017 [41]	USA	116 participants (96 analysed)<br><br>30-54 years<br><br>Mean age: 41.38 years (7.57)<br><br>80% female<br><br>Low-active	12-week, 4-group randomised factorial trial<br><br>Pre-intervention assessments: 1-week	*Mobile application*<br>Base application (newly developed):<br>Tracking; instant feedback; bi-weekly feedback<br>Four versions of the base application with additional features:<br>1. Goal setting & points<br>2. Goal setting<br>3. Points<br>4. Base app only	*Pre-intervention & 12 weeks*<br><br>Actigraph accelerometer (worn on 7 consecutive days)<br>Average daily minutes of MVPA	*Pre-intervention & 12 weeks*<br><br>The exercise self-efficacy scale<br><br>Barriers specific self-efficacy scale<br><br>Perceived barriers to exercise	*Physical activity outcomes*<br><br>Significant increase in MVPA across the intervention from baseline (M = 34.88, SD = 1.62) to 12 weeks (M = 46.77, SD = 1.65) (*P* < .01, *d* = 0.70)<br><br>*Psychosocial outcomes*<br><br>Significant decrease in perceived barriers to exercising across the	Social Cognitive Theory

				Intervention<br>Initial in-person session on goal setting; provided with one of the four versions of the application; wore Actigraph accelerometer		Outcome expectations for exercise<br><br>App engagement<br>Recorded by app (date and time app was opened)	intervention from baseline (M = 62.38, SD = 0.87) to 12 weeks (M = 54.54, SD = 1.10) (*P* = .01, *d* = 0.73)<br><br>No significant changes in exercise self-efficacy; (*P* = .12, *d* = -0.24), barriers specific self-efficacy; (*P* = .11, *d* = -0.14), and outcome expectations (*P* = .34, *d* = 0.04)<br><br>*Intervention engagement*<br><br>App use significantly decreased across the intervention period (*P* < .01)	
Glynn et al., 2014 [36]	Ireland	90 participants (77 analysed)<br><br>Mean age: 44.1 years (11.5)<br><br>64% female<br><br>Primary care	8-week, 2 group RCT<br><br>Pre-intervention assessments: 1-week	*Mobile Application*<br>Accupedo-Pro Pedometer App (commercially available):<br>Tracking of daily step count and calories burnt; automatic feedback; step history; and goal achievement<br><br>*Intervention condition (n = 45)*<br>Provision of the application on phone; instructions on how to use application; given physical activity goal (10, 000 steps per day); information on benefits of exercise<br><br>*Control condition (n = 45)*<br>Provision of the application on phone; application not made visible on phone and no instructions on how to use app; given physical activity goal	*Pre-intervention & 8 weeks*<br><br>Accupodo-Pro Pedometer App<br>Daily step count		*Physical activity outcomes*<br><br>Significant between-group difference in daily step count, *P* = .02<br>Intervention condition; baseline (M = 4365, SD = 3873); post-intervention (M = 5855, SD = 4264) (*d* = 0.37)<br>Control condition; baseline (M = 5138, SD = 3873); post-intervention (M = 4859, SD = 3474) (*d* = 0.07)	Not reported

				(walking for 30 minutes per day); information on benefits of exercise				
Korinek et al., 2018 [50]	USA	20 participants<br><br>40-65 years<br><br>Mean age: 47.25 years (6.16)<br><br>90% female<br><br>Overweight (BMI of 25-45 kg/m^2) & insufficiently active	14-week within-subject, pre-post design<br><br>Pre-intervention assessments: 2 weeks	*Mobile Application*<br>Just Walk Application (newly developed):<br>Adaptive step goals; points received for achieving daily steps goals; monitoring of progress<br><br>*Intervention*<br>Received Just Walk Application; wore Fitbit Zip	*Pre-intervention & 14 weeks*<br><br>Fitbit Zip<br>Daily step count		*Physical activity outcomes* (M & SD not reported)<br><br>A significant increase in daily steps ($P < .01$)<br><br>Insufficient data to calculate effect sizes	Social Cognitive Theory
Pellegrini et al., 2015 [49]	USA	9 participants (8 analysed)<br><br>21-70 years<br><br>Mean age: 53.1 years (10.7)<br><br>77% female<br><br>Type 2 diabetes & sedentary lifestyle	1-month within-subject, pre-post design<br><br>Pre-intervention assessments: 10-12 days	*Mobile Application*<br>NEAT! application (newly developed):<br>Prompts (noise or vibration) to stand after 20 minutes of consecutive sedentary time<br><br>*Intervention*<br>Received NEAT! Application; wore Actigraph accelerometer	*Pre-intervention & 1-month*<br><br>Actigraph Accelerometer (worn on 10 consecutive days)<br>Sedentary behaviour, light physical activity, and MVPA	*Over 1-month intervention period*<br><br>App engagement<br>Days and hours of usage	*Physical activity outcomes*<br>(M & SD not reported for $n = 8$)<br><br>No significant decrease in sedentary time ($P = .08$)<br><br>Significant increase in light physical activity ($P = .04$)<br>No significant changes in MVPA (*P*-value not reported)<br><br>*Intervention engagement*<br><br>Used app on 21.9 (8.0) days for 7.6 (2.5) hours<br><br>Insufficient data to calculate effect sizes	Not reported

Simons et al., 2018 [42]	United Kingdom	130 participants<br><br>18-30 years<br><br>Mean age: 25 years (3.0)<br><br>51.5% female<br><br>Low-active	9-week, 2 group RCT<br><br>Pre-intervention assessments: 1-week<br><br>Follow-up assessments: 3 months post-intervention	*Mobile Application*<br><br>Active Coach app (newly developed):<br>9-week program; tailored personal goals; feedback on goal achievement; tips and facts provided through notifications to encourage physical activity; self-monitoring of steps<br><br>*Intervention condition (n = 60)*<br>Received Active Coach app; wore Fitbit Charge<br><br>*Control condition (n = 70)*<br>Did not receive app; brochure with information and tips to encourage physical activity	*Pre & post-intervention & follow-up*<br><br>Actigraph accelerometer (worn on 7 consecutive days); Light, moderate and vigorous physical activity, MVPA, and steps<br><br>International Physical Activity Questionnaire (IPAQ);<br>Frequency and duration of occupational physical activity, active transport, household physical activity and recreational physical activity	*Pre & post- intervention & follow-up*<br><br>Face-to-face interview<br>Psychosocial variables; social support, attitude (perceived benefits and barriers) and self-efficacy<br><br>App engagement<br>Google Analytics to obtain number and duration of app visits, and monitoring of frequency of reading messages relating to goals, tips and facts	*Physical activity outcomes*<br><br>No significant group by time interaction for objective measures of physical activity including, light physical activity (*P* = .31), moderate physical activity (*P* = .56), vigorous physical activity (*P* = .26), MVPA (*P* = 0.66), steps per day (*P* = .64)<br><br>No significant group by time interaction for self-reported measures of physical activity including, occupational physical activity (*P* = .88), active transport (P = .98), household physical activity (*P* = .52) and recreational physical activity (*P* = .84)<br><br>*Psychosocial outcomes*<br><br>No significant group by time interaction for perceived benefits (*P* = .75), perceived barriers (*P* = .82), self-efficacy (*P* = .41) and social support, (*P* = .25)<br><br>*Intervention Engagement*<br><br>The number of visits halved from 824 visits in the first 3-weeks to 403 visits in the last 3-weeks<br><br>The average duration of visiting the app was 1:19 minutes in the first 3-weeks, and 53 seconds in the last 3-weeks	Attitude-social influence self-efficacy model

							Significant decrease in frequency of reading the messages in relation to goals (*P* < .01), tips and facts (*P* = .49)<br><br>Insufficient data to calculate effect sizes	
Walsh et al., 2016 [37]	Ireland	55 participants<br><br>17-26 years<br><br>Mean age: 20.55 years (2.07)<br><br>72.7% female	5-week, 2 group RCT<br><br>Pre-intervention assessments: 1-week	*Mobile Application*<br>Accupodo-Pro Pedometer App (commercially available): Tracking of daily step count and calories burnt; automatic feedback; step count history; goal-setting functionality; and goal achievement feedback<br><br>*Intervention condition (n* = 28*)*<br>Received Accupodo-Pro Pedometer App; instructions on how to use application; given physical activity goal (10, 000 steps per day); information on benefits of exercise<br><br>*Control condition (n* = 27*)*<br>Received Accupodo-Pro Pedometer App; application not made visible on phone and no instructions on how to use app; given physical activity goal (walking for 30 minutes per day); information on benefits of exercise	*Pre-intervention & 5 weeks*<br><br>Accupodo-Pro Pedometer App Daily step count		*Physical activity outcomes*<br>(M & SD not reported)<br><br>Significant between-group difference in daily step count, F(1, 53) = 4.30, *P* = .04, η_p^2 = 0.08<br><br>Significant increase in step counts for from baseline to follow-up for both intervention condition, *t*(27) = -6.14, *P* <.001 and control condition, *t*(26) = -2.25, *P* = .03<br><br>Significantly higher increase in step count in the intervention condition (2393 steps) than the control condition (1101 steps), *t*(53) = 2.07, *P* = .04	Capability, Motivation, Behaviour (COM-B) framework/ Behaviour Change Wheel
