# Supplementary material for: A Comparison of Physical Activity Mobile Apps With and Without Existing Web-Based Social Networking Platforms: Systematic Review
Source: J Med Internet Res. 2019 Aug 16;21(8):e12687. doi: 10.2196/12687 (PMC6716337; doi:10.2196/12687)
Supplement: Multimedia Appendix 4 [file jmir_v21i8e12687_app4.pdf]

Reference	Country	Sample Characteristics	Study Design	Description of Intervention	Features of Existing Web-based Social Network	Physical Activity Outcome Measure(s)	Other Measures	Key Findings	Behaviour Change Theory
Al Ayubi et al., 2014 [43]	USA	13 participants<br><br>18-65 years<br><br>Mean age: 32.15 years<br><br>76.92% female	4-week, within-subject pre-post design<br><br>No social interaction: week 1<br>Social interaction enabled: week 2	*Mobile Application*<br>Persuasive Social Network for Physical Activity Application (PersonA) (newly developed): Accelerometer to measure physical activity; goal setting; self-monitoring feedback on progress; peer comparison (compare performance with one other person and group average)<br><br>*Intervention*<br>Received PersonA	PersonA links to Facebook: Share physical activity data; post, like, comment on data; communicate	*Pre-intervention & 4 weeks*<br><br>PersonA accelerometer<br>Number of steps	*Pre-intervention & 4 weeks*<br><br>App engagement<br>PersonA recorded minutes of use	*Physical activity outcomes*<br><br>Average number of steps increased from 4202 at baseline (no social interaction) to 6352 steps following the enablement of social interaction (*P*- value not reported)<br><br>*Intervention engagement*<br><br>The duration of system use increased from 419 minutes at baseline, to 465 minutes in week 2.<br><br>Insufficient data to calculate effect sizes	Health Belief Model;<br>Theory of reasoned action/ theory of planned behaviour;<br>Elaboration Likelihood Model;<br>Social Cognitive theory; Social supportive and health link theory;<br>Users and gratification theory;<br>Common bond and common identity theory;<br>Technology Acceptance Model;<br>Unified theory of acceptance and use of technology;<br>Fogg Behavioural Model
Foster et al., 2010 [44]	United Kingdom	10 participants<br><br>Age range: not specified<br><br>Mean age: not specified<br><br>90% female	21-days within-subject pre-post design, randomised cross-over<br><br>Pre-intervention assessments:	*Mobile Application*<br><br>Step Matron Application (newly designed): Monitoring of daily steps<br><br>*Intervention*<br>All participants engaged in the two conditions:	Facebook<br><br>View each other's step data, make comments and comparisons (rankings table)	*Pre-intervention & 5 days*<br><br>Pedometer<br>Number of steps	*Pre-intervention & 5 days*<br><br>Google Analytics<br>App logins and minutes of use	*Physical activity outcomes*<br><br>Significantly higher number of steps when participants used the social condition (M = 42002, SD = 7040) than the non-social condition (M = 38132.1, SD = 7800) (*P* = .01, *d* = 0.52)	Not reported

		Registered nurses	specific details not provided	1. Socially enabled condition<br>Received Step Matron Application; access to Facebook; wore pedometer<br>2. Non-socially enabled condition<br>Received Step Matron Application; wore pedometer				*Intervention engagement*<br><br>1:46 minutes engaging with app during non-social condition<br>2:37 minutes engaging with app during social enabled condition	
Hurkmanns et al., 2018 [47]	United Kingdom	102 participants (81 analysed)<br><br>18-65 years<br><br>Mean age: 45 years (10.35)<br><br>69.7% female<br><br>Overweight & obese (29 and 34 kg/m[2])	12-week, 4-group RCT<br><br>Pre-intervention assessments: specific details not provided	*Mobile Application*<br><br>Mobile weight loss application (newly developed):<br>Advice on dietary patterns and physical activity; tracking of step count; self-monitoring; information on nutrition and physical activity; links to Facebook group<br><br>*Intervention Conditions*<br><br>*1.* Conventional condition; Individualised diet plan from a dietician; individualised physical activity plan; access to a dietician (week 1, 2 & 5); access to a physical activity coach (week 1, 2, 5 & 7)<br>2. App condition;	Facebook Group	*Pre-intervention & 12 weeks*<br><br>Tri-axial accelerometer (ActiGraph)<br>Time spent in moderate-to-vigorous physical activity (MVPA)		*Physical activity outcomes*<br><br>No significant group by time interaction effects for MVPA (*P*- value not reported)	Not reported

				Access to mobile application<br>3. Combination condition; Access to a dietician (week 1); access to a physical activity coach (week 1 & 7); access to mobile weight loss app<br><br>*Control Condition*<br>Wait list control					
Pope et al., 2018 [46]	USA	10 participants<br>≥ 21 years<br>Mean age: 45.80 years (10.23)<br>100% female<br>Breast cancer survivors (no contraindications to physical activity)	10-week, within-subject pre-post design<br>Pre-intervention assessments: 7 days<br>Follow-up assessments: 1-week post-intervention	*Mobile Application*<br>MapMyFitness Application (commercially available): Day to day physical activity diary<br>*Intervention*<br>MapMyFitness Application; Facebook page	Facebook Page: Education tips based on Social Cognitive Theory posted to page twice a week: Encouraged to post/ comment on the page	*Pre-intervention & follow-up*<br>Accelerometer (worn on 7 consecutive days) Average daily minutes of sedentary behaviour, light physical activity and MVPA	*Pre-intervention & follow-up*<br>Self-efficacy Scale<br>Patient-centred Assessment and Counselling Questionnaire Social support<br>Physical activity enjoyment Scale<br>*Midpoint and post-intervention*<br>Intervention engagement<br>Self-report survey of MayMyFitness usage; frequency/duration of use	*Physical activity outcomes*<br>Increase in average daily steps; baseline (M = 4930, SD = 1376); post-intervention (M = 6587, SD = 1229) (*d* = 1.27), and average daily MVPA; baseline (M = 26.8, SD = 13.8); post-intervention (M = 29.4, SD = 22.5) (*d* = 0.14)<br>Decrease in average daily light physical activity from baseline (M = 94.9, SD = 44.8) to post-intervention (M = 86.7, SD = 64.7) (*d* = 0.15), and average daily sedentary behaviour; baseline (M = 493.7, SD = 176); post-intervention (M = 381, SD = 265.3) (*d* = 0.50)	Social Cognitive Theory

							Engagement with Facebook; posts generated and viewed	*Psychosocial outcomes*<br><br>Increases in social support; baseline (M = 2.82, SD = 0.92); post-intervention (M = 3.38, SD = 1.24) (*d* = 0.51), self-efficacy; baseline (M = 72.89, SD = 29.70); post-intervention (M = 75.28, SD = 25.74) (*d* = 0.09), and enjoyment to exercise; baseline (M = 3.18, SD = 0.90); post-intervention (M = 3.33, SD = 0.80) (*d* = 0.13)<br><br>*Intervention engagement*<br><br>Frequency of MapMyFitness use; midpoint used 3.75 times per week; post-intervention used 4.34 times per week<br>Duration of MayMyFitness use;<br>midpoint used for 39.7minutes per week; post-intervention used 35 minutes per week<br><br>93% of participants viewed each Facebook post	
Torquati, Kolbe-Alexander et al. 2018 [45]	Australia	47 participants<br><br>>18 years	3-month, within-subject pre-post design	*Mobile Application*<br><br>Smartphone application (newly designed):	Private Facebook group<br>Posting of motivational and inspirational	*Pre-intervention, 3 months & follow-up*	*Pre-intervention, 3 months & follow-up*<br><br>Social support scale	*Physical activity outcomes*<br><br>Significant decrease in percentage of daily time spent in MVPA; baseline (M	Social Cognitive Theory;<br>Goal-setting Theory;<br>Control Theory

		Mean age: 41.4years (12.1)<br><br>87% female<br><br>Nurses	Pre-intervention assessments: 7 days<br><br>Follow-up assessments: 3 months post-intervention	Facilitate physical activity and diet goal-setting<br><br>*Intervention* Smartphone application; Facebook Group; wore Pedometer	quotes to be active/healthy	Accelerometer (worn on 7 consecutive days) MVPA, daily steps, sedentary behaviour and light physical activity	Physical activity self-efficacy scale<br><br>Intervention engagement<br><br>Recorded use of intervention content; Viewing of pedometer and application instructions; views of Facebook posts	= 3.0, SD = 1.9); 3 months (M = 2.5, SD = 1.9); 6 months (M = 2.5, SD = 2.0) (*P* = .01, *d* = 0.26), and daily average steps; baseline (M = 8496, SD = 2528); 3 months (M = 8136, SD = 2395), 6 months (M = 7629, SD = 2342) (*P* = .05, *d* = 0.15)<br><br>No significant changes in sedentary behaviour (P = .70) or light physical activity (P = .56).<br><br>*Psychosocial outcomes* (*P* values not reported)<br><br>No significant changes in self-efficacy or social support<br><br>*Intervention engagement*<br><br>68.4% used app less than once a month or never<br><br>47.4% engaged with Facebook group at least once	
