# Supplementary material for: A Comparison of Physical Activity Mobile Apps With and Without Existing Web-Based Social Networking Platforms: Systematic Review
Source: J Med Internet Res. 2019 Aug 16;21(8):e12687. doi: 10.2196/12687 (PMC6716337; doi:10.2196/12687)
Supplement: Multimedia Appendix 6 [file jmir_v21i8e12687_app6.pdf]

Checklist Item	Al Ayubi, Parmanto et al. 2014 [43]	Foster, Linehan et al. 2010 [44]	Hurkmans, Matthys et al. 2018 [47]	Pope, Lee et al. 2018 [46]	Torquati, Kolbe-Alexander et al. 2018 [45]
**<u>Title and Abstract</u>**					
**1.** a) Identification as randomized trial in the title; b) structured summary of trial design, methods, results, and conclusions	--<br>✓	--<br>✓	✓<br>✓	--<br>✓	--<br>✓
**<u>Introduction</u>**					
**2.** a) Scientific background and explanation of rationale; b) specific objectives/hypotheses	✓<br>✓	✓<br>✓	✓<br>✓	✓<br>✓	✓<br>✓
**<u>Methods</u>**					
**3. Trial Design**<br>a) Description of trial design; b) important changes to methods after trial commencement, with reasons	✓<br>--	✓<br>--	✓<br>✓	✓<br>--	✓<br>--
**4. Participants**<br>a) Eligibility criteria for participants; b) settings and locations of data collection	✓<br>✓	✓<br>--	✓<br>✓	✓<br>✓	✓<br>✓
**5. Intervention**<br>The interventions for each group with sufficient details to allow for replication, including how and when they were actually administered	✓	✓	✓	✓	✓
**6**. **Outcomes**<br>a) Completely defined pre-specified primary and secondary outcome measures, including how and when they were assessed; b) any changes to trial outcomes after trial commenced, with reasons	✓<br>--	✓<br>--	✓<br>--	✓<br>--	✓<br>--
**7. Sample Size**<br>a) How sample size was determined; b) when applicable explanation of any interim analysis and stopping guidelines	✓<br>--	--<br>--	✓<br>--	--<br>--	--<br>--

	Al Ayubi, Parmanto et al. 2014 [43]	Foster, Linehan et al. 2010 [44]	Hurkmans, Matthys et al. 2018 [47]	Pope, Lee et al. 2018 [46]	Torquati, Kolbe-Alexander et al. 2018 [45]
**8. Randomisation: Sequence Generation**<br>a) Method used to generate the random allocation sequence; b) type of randomisation including details of any restriction	--<br>--	--<br>--	✓<br>✓	--<br>--	--<br>--
**9. Allocation Concealment Mechanism**<br>Mechanism used to implement the random allocation sequence, describing any steps taken to conceal sequence until interventions were assigned	--	--	--	--	--
**10. Implementation**<br>Who generated the random allocation sequence, who enrolled participants, and who assigned participants to the interventions	--	--	✓	--	--
**11. Blinding**<br>a) If done, who was blinded after assignment to interventions and how; b) if relevant, similarity of interventions	--<br>--	--<br>--	✓<br>--	--<br>--	--<br>--
**12. Statistical Methods**<br>a) Statistical methods used to compare groups for primary and secondary outcomes; b) methods for additional analyses, such as subgroup analyses and adjusted analyses	--<br>--	✓<br>--	✓<br>--	✓<br>--	✓<br>✓
<u>**Results**</u>					
**13. Participant flow**<br>a) For each group, the numbers of participants who were randomly assigned, received intended treatment, and were analysed for the primary outcome; b) for each group losses and exclusions after randomisation, with reasons	✓<br>--	✓<br>--	✓<br>✓	✓<br>✓	✓<br>✓
**14. Recruitment**<br>a) Dates defining the periods of recruitment and follow-up; b) why the trial ended or was stopped	--<br>--	--<br>--	✓<br>--	✓<br>--	--<br>--

	Al Ayubi, Parmanto et al. 2014 [43]	Foster, Linehan et al. 2010 [44]	Hurkmans, Matthys et al. 2018 [47]	Pope, Lee et al. 2018 [46]	Torquati, Kolbe-Alexander et al. 2018 [45]
**15. Baseline Data**<br>A table with baseline demographic and clinical characteristics for each group	✓	--	✓	✓	✓
**16. Numbers Analysed**<br>For each group, number of participants included in each analysis and whether the analysis was by original assigned groups	✓	✓	✓	✓	✓
**17. Outcomes and Estimation**<br>a) For each primary and secondary outcome, and the estimate effect sizes and its precision; b) for binary outcomes presentation of both absolute and relative effect sizes is recommended	--<br>--	✓<br>--	--<br>--	--<br>--	--<br>--
**18. Ancillary Analyses**<br>Results of any other analyses performed, including subgroup analyses and adjusted analyses, distinguishing pre-specified from exploratory	--	--	--	--	✓
**19. Harms**<br>All-important harms or unintended effects in each group	--	--	--	--	--
**<u>Discussion</u>**					
**20. Limitations**<br>Trial limitations, addressing sources of potential bias, imprecision, and, if relevant, multiplicity of analyses	✓	--	✓	✓	✓
**21. Generalisability**<br>Generalisability (external validity, applicability) of the trial findings	✓	--	✓	✓	✓
**22. Interpretation**<br>Interpretation consistent with results, balancing benefits and harms, and considering other relevant evidence	✓	✓	✓	✓	✓
**<u>Other Information</u>**					
**23. Registration**<br>Registration number and name of registry	--	--	✓	--	--

	Al Ayubi, Parmanto et al. 2014 [43]	Foster, Linehan et al. 2010 [44]	Hurkmans, Matthys et al. 2018 [47]	Pope, Lee et al. 2018 [46]	Torquati, Kolbe-Alexander et al. 2018 [45]
**24. Protocol**<br>Where the full trial protocol can be accessed	--	--	--	--	--
**25. Funding**<br>Sources of funding/role of funders	✓	✓	✓	✓	--
**Total**	11.5	8.5	18	12.5	12.5

[a] Criterion fulfilled: ✓; Criterion not fulfilled: --
